# Supplementary figures and images for: Two distinct lipid transporters together regulate invasive filamentous growth in the human fungal pathogen Candida albicans
Source: PLoS Genet. 2022 Dec 14;18(12):e1010549. doi: 10.1371/journal.pgen.1010549 (PMC9797089; doi:10.1371/journal.pgen.1010549)

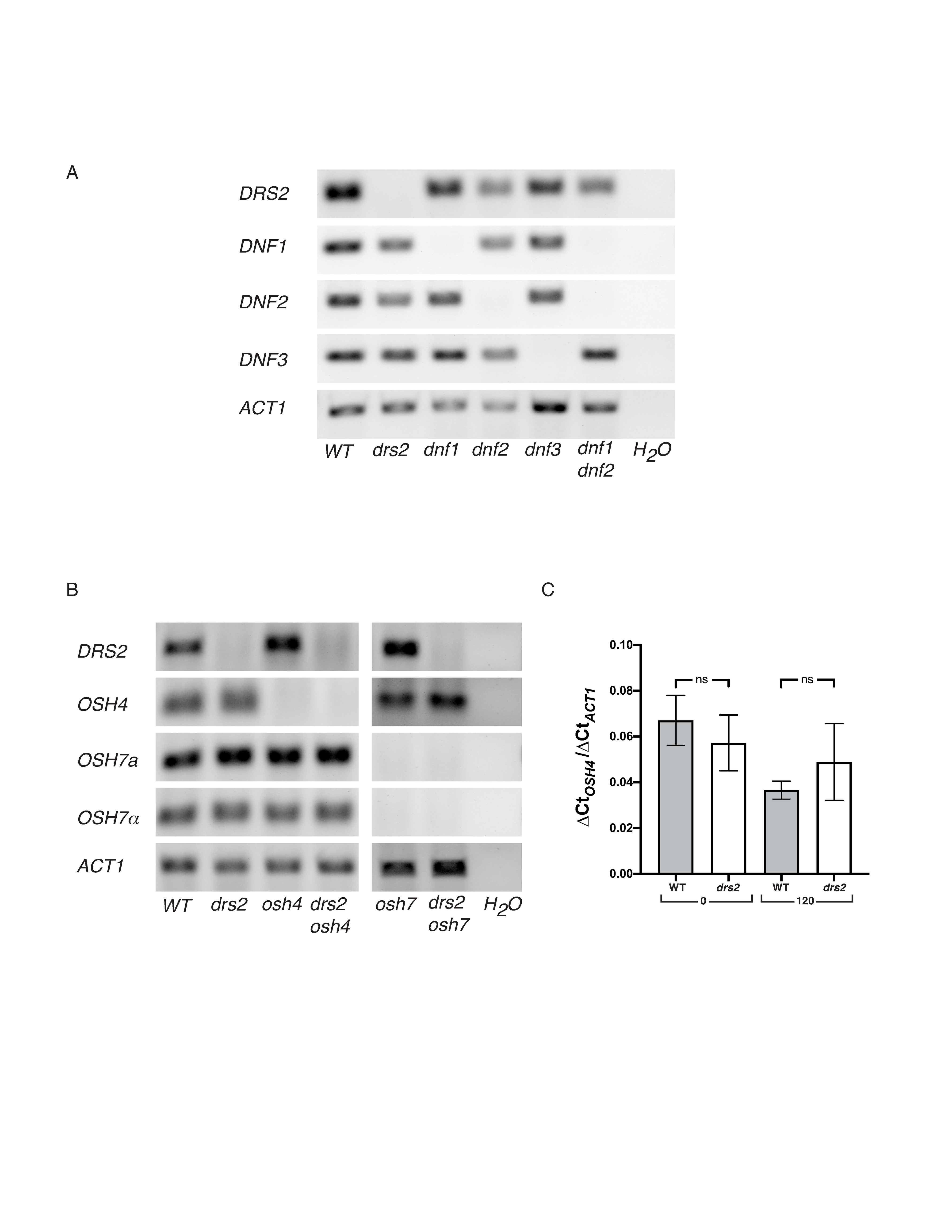

Supplement: S1 Fig — A) DRS2 and DNF1-3 transcript levels. mRNA and cDNA were prepared from the indicated strains, as in Fig 1A. DRS2 and DNF1-3 transcripts were determined by RT-PCR, using DRS2.pTm/DRS2.mTm (62 bp), DNF1.pTm/DNF1.mTm (73 bp), DNF2.pTm/DNF2.mTm (106 bp) and DNF3.pTm/DNF3.mTm (104 bp) primer pairs, respectively. Actin (ACT1) transcript levels (ACT1.pTm/ACT1.mTm primer pair) were used for normalization. B) OSH4 and OSH7 transcript levels. mRNA and cDNA were prepared from the indicated strains, as in Fig 8A. Transcripts were determined as in S1A Fig, using OSH4.pTm/OSH4.mTm (69 bp), OSH7A.pTm/OSH7A.mTm (84 bp), OSH7α.pTm/ OSH7α.mTm (65 bp) primer pairs, respectively. C) Deletion of DRS2 does not result in a significant alteration of OSH4 expression. mRNA and cDNA were prepared from the indicated strains grown without (0) or with serum for 120 min (120). OSH4 and DRS2 transcripts were determined by qRT-PCR using primer pairs OSH4.pTm/OSH4.mTm and DRS2.pTm/DRS2.mTm as above. Bars indicate the mean ± SD of 2 biological samples (n = 3 determinations each). ACT1 transcript levels were used for normalization. (TIF) [file pgen.1010549.s001.tif]

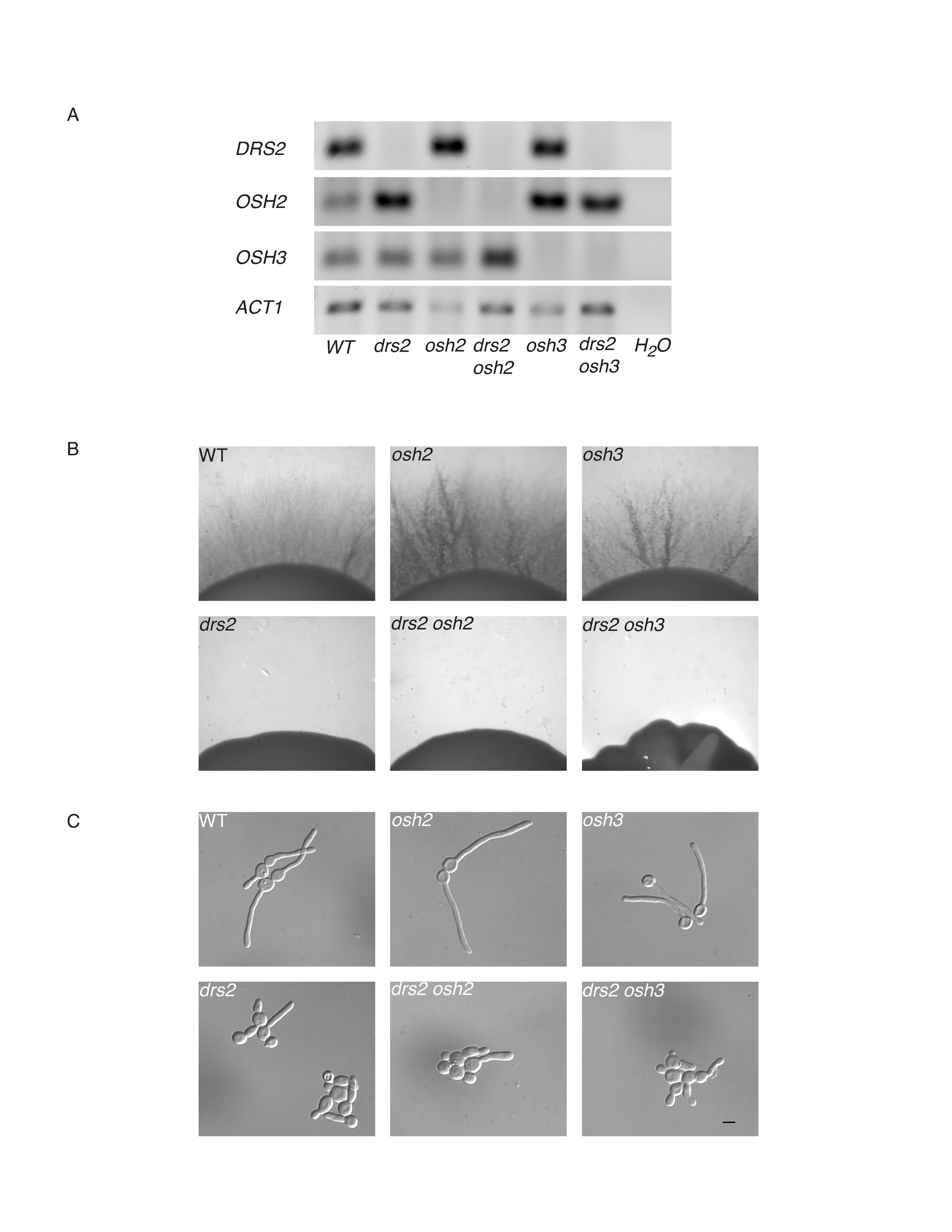

Supplement: S2 Fig — A) DRS2 and OSH2-3 transcript levels. mRNA and cDNA were prepared from the indicated strains, and transcripts were determined as in S1A Fig, using OSH2.pTm/OSH2.mTm (74 bp), OSH3.pTm/OSH3.mTm (74 bp) primer pairs, respectively. B) Invasive growth is not restored in the drs2 mutant upon deletion of OSH2 or OSH3. The indicated strains, WT (PY4861), osh2/osh2 (osh2, PY3977), osh3/osh3 (osh3, PY4002), drs2/drs2 (drs2, PY3375), drs2/drs2 osh2/osh2 (drs2 osh2, PY6408) and drs2/drs2 osh3/osh3 (drs2 osh3, PY6431), were grown on agar-containing serum media and images were taken after 6 days. Similar results were observed in 2 independent experiments. C) Hyphal growth is not restored in the drs2 mutant upon deletion of OSH2 or OSH3. At 90 min, the percent of filamentous cells, was determined from 3 independent biological samples for the WT, drs2, osh2, osh3, drs2 osh2 and drs2 osh3 strains; n ~ 100 cells. (TIF) [file pgen.1010549.s002.tif]
